# Berberine Attenuates Experimental Autoimmune Encephalomyelitis in C57 BL/6 Mice

**DOI:** 10.1371/journal.pone.0013489

**Published:** 2010-10-19

**Authors:** Xiaomeng Ma, Ying Jiang, Aimin Wu, Xiaohong Chen, Rongbiao Pi, Mei Liu, Yingying Liu

**Affiliations:** 1 Department of Neurology, The Third Affiliated Hospital, Sun Yat-sen University, Guangzhou, China; 2 Department of Pharmacology and Toxicology, School of Pharmaceutical Sciences, Sun Yat-sen University, Guangzhou, China; University of New South Wales, Australia

## Abstract

**Background:**

Berberine, an isoquinoline derivative alkaloid, has a wide range of pharmacological properties and is considered to have anti-inflammatory and neuroprotective effects. However, there are no reports about the effects and mechanisms of berberine in experimental autoimmune encephalomyelitis (EAE), an established model of multiple sclerosis (MS).

**Methodology/Principal Findings:**

Female C57 BL/6 mice immunized with myelin oligodendrocyte glycoprotein 35–55 amino acid peptide were treated with berberine at the day of disease onset and medication was administered daily until mice were sacrificed. Blood–brain barrier (BBB) permeability and the alteration of matrix metalloproteinase-2 (MMP-2, 72 kDa) and matrix metalloproteinase-9 (MMP-9, 92 kDa) in the brain and cerebrospinal fluid (CSF) of EAE mice were detected by quantitative measurement for Evan's blue (EB) content, Western blot and gelatin zymography respectively. The results showed that berberine attenuated clinical and pathological parameters of EAE, reduced the permeability of BBB, inhibited the activity and expression of MMP-9 but not MMP-2 in the CSF and brain of EAE mice.

**Conclusions/Significance:**

These findings suggest that berberine is effective to attenuate the clinical severity of EAE in C57 BL/6 mice by reducing the permeability of BBB, decreasing the expression and activity of MMP-9, and decreasing the inflammatory infiltration. We think that berberine might be a potential therapeutic agent for MS.

## Introduction

Multiple Sclerosis (MS) is a chronic inflammatory demyelinating autoimmune disease of the central nervous system (CNS) which classically exhibits a relapsing- remitting course with increasing neurological sequelae [1]. Experimental autoimmune encephalomyelitis (EAE), a T-cell-mediated model of autoimmune demyelination of the CNS, is an established model of MS that has been utilized to test novel therapies [2]. Dysfunction of the blood–brain barrier (BBB) and migration of T lymphocytes into the CNS are the hallmarks in the pathogenesis of MS and EAE [3]. The entry of leukocytes into the CNS is dependent on several factors, most likely including the expression of matrix metalloproteinases (MMPs) [4], [5].

Berberine is an isoquinoline derivative alkaloid isolated from many medicinal herbs, such as Hydrastis canadensis (goldenseal), Cortex phellodendri (Huangbai) and Rhizoma coptidis (Huanglian) [6]. It is frequently utilized in proprietary Chinese herbal drugs to treat inflammation, especially in the oral cavity [7]. Besides its anti-inflammation effects, many other pharmacological benefits of berberine have also been reported including anti-oxidation, anti-tumor and neuroprotective effects [8]–[12], but these effects were strongly correlated with its anti-inflammatory properties [7], [11]. It has been reported that berberine could play its anti-inflammatory effects through inhibiting the production of tumor necrosis factor-α (TNF-α), interleukin-6 (IL-6) and monocyte chemoattractant protein 1 (MCP-1), suppressing cyclooxygenase-2 (COX-2) expression, retarding prostaglandin E_2_ (PGE_2_) production and exudates formation, and down-regulating the expression of matrix metalloproteinase-2 (MMP-2) and matrix metalloproteinase-9 (MMP-9) through mitogen-activated protein kinase (MAPK) and nuclear factor-κB (NF-κB) signaling pathways [7], [13], [14], [15]. However, there are no reports about the effects of berberine on the immune inflammatory diseases of CNS, such as MS.

In this study, we tested whether berberine was effective to ameliorate the clinical severity in C57 BL/6 EAE mice induced by myelin oligodendrocyte glycoprotein 35–55 amino acid peptide (MOG35–55). We further investigated the permeability of BBB and the alteration of MMP-2 (72 kDa) and MMP-9 (92 kDa) in the cerebrospinal fluid (CSF) and the brain of EAE mice. Our results demonstrated that berberine could attenuate clinical and pathological parameters of EAE, reduce the permeability of BBB, inhibit the activity and expression of MMP-9 but not MMP-2 in the CSF and brain in EAE mice.

## Results

### Berberine ameliorated clinical severity of EAE mice

**Figure 1:**
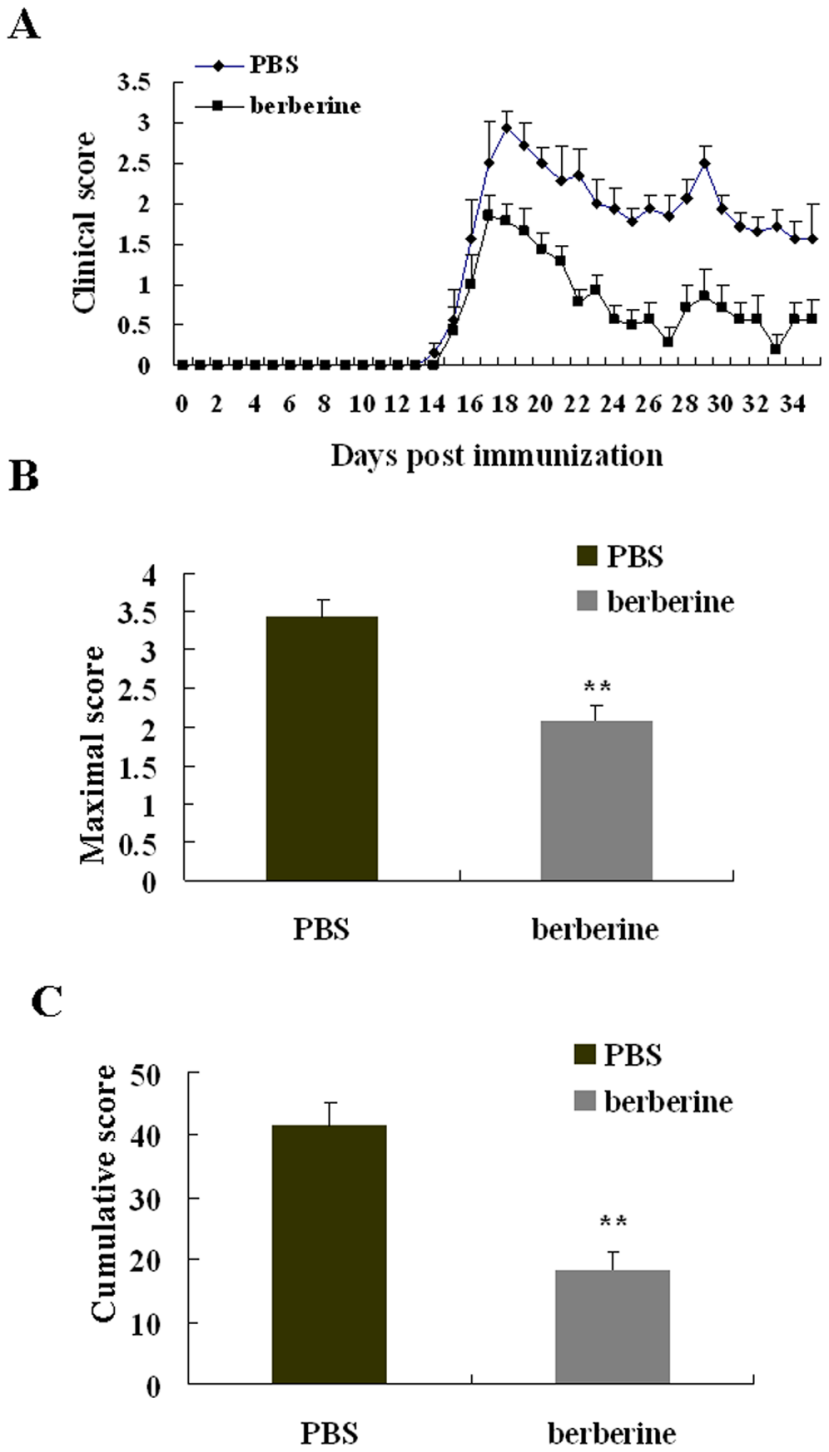
The clinical course of EAE was attenuated by berberine treatment. Daily clinical score (A), maximal score (B) and cumulative score (C) of the PBS- and berberine-treated EAE mice. EAE was induced in female C57 BL/6 mice with MOG 35-55 and treatment with berberine was performed as described under materials and methods. Each group had seven animals. Drug treatments were started from the day of disease onset. Values represent the mean±S.E.M., **P<0.01 vs. PBS-treated EAE mice.

EAE was induced in six to eight-week-old female C57 BL/6 mice by immunization with MOG 35-55 as previously described [16]. Berberine was intragastric administration via per os and the therapy was started at the disease onset and medication was administered daily until mice were sacrificed at day 35 post immunization (p.i.). We found that the day of disease manifestation and the severity of symptoms at disease manifestation were similar in the phosphate buffered saline (PBS)-treated and berberine-treated groups (each group has seven animals, Fig. 1A). The maximal score (maximum severity of disease in individual mouse) of PBS- and berebrine-treated EAE mice were 3.43±0.22 and 2.10±0.22 respectively. The cumulative score (cumulative disease severity) of PBS- and berbreine-treated EAE mice were 41.50±3.75 and 18.36±2.83 respectively. The maximal score and the cumulative score were significantly reduced in berberine-treated EAE mice (P<0.01, Fig. 1 B,C).

### Berberine improved neuropathology of EAE mice

**Figure 2:**
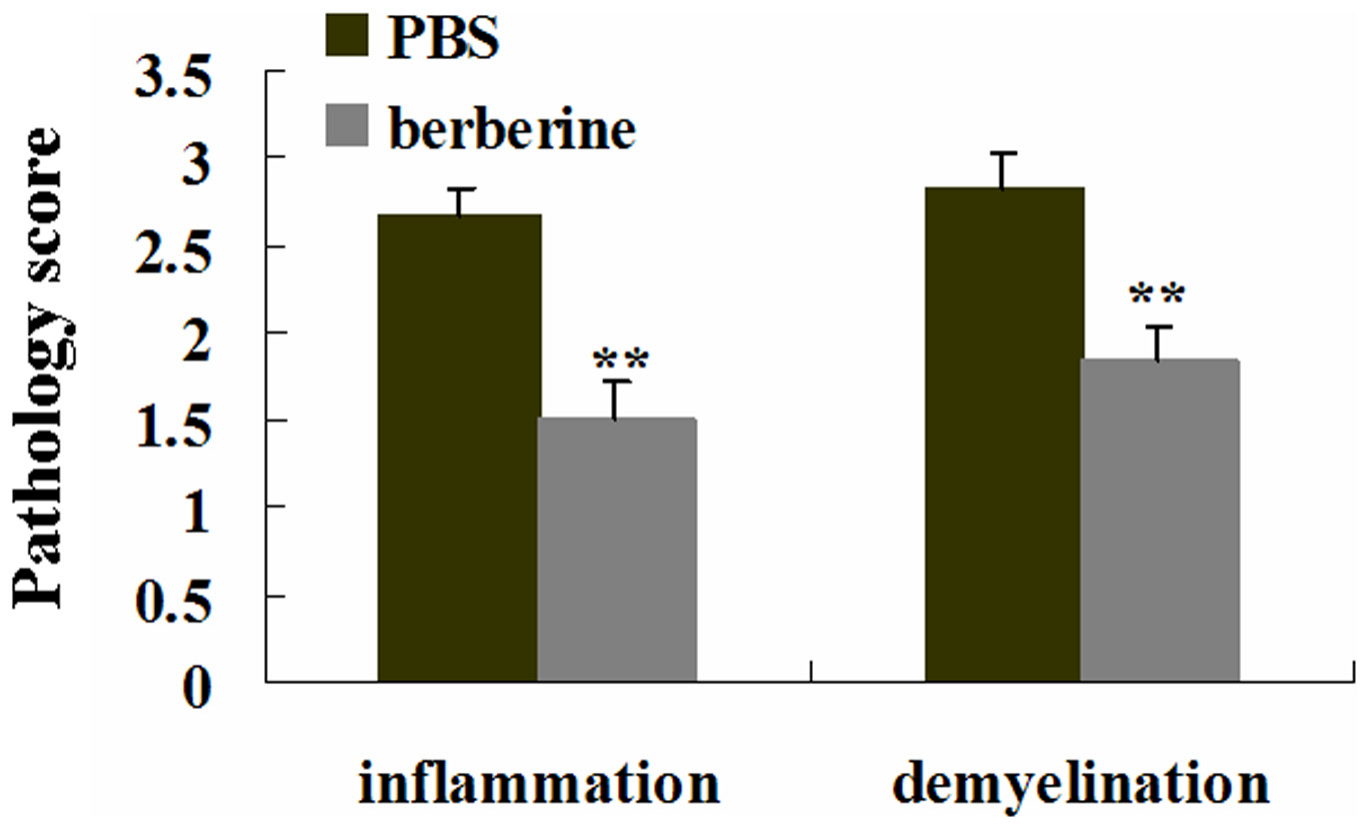
Berberine treatment decreased inflammation and demyelination in the lumbar spinal cord of EAE mice. Mice were subjected to histopathological assay at day 20 p.i.. Each group had six animals. Consecutive sections were analyzed for hematoxylin and eosin staining and solochrome cyanin staining to detect inflammatory infiltration and demyelination respectively. Values represent the mean±S.E.M., **P<0.01 vs. PBS-treated EAE mice.

**Figure 3:**
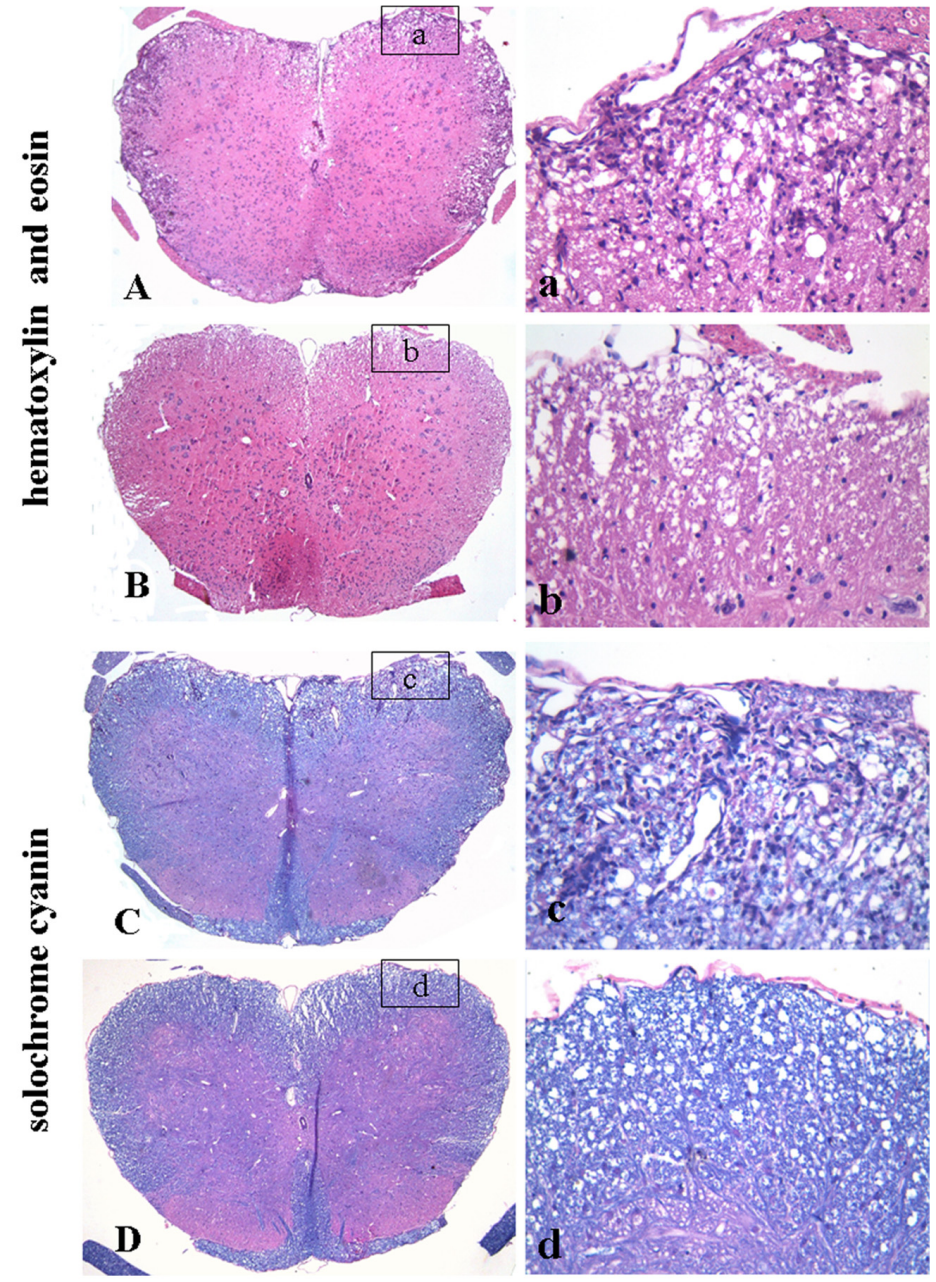
The representative sections depicting the inflammation and demyelination in the lumbar spinal cord of EAE mice by PBS and berberine treatment. Inflammatory infiltration was widespread in the PBS-treated EAE mice (A), but which was attenuated by berberine treatment (B). A large plaque of demyelination was seen in the PBS-treated EAE mice (C), and demyelination was attenuated in berberine-treated EAE mice (D). a-d were higher magnification views of the boxed areas in A, B, C and D, respectively. A-D: ×40; a-d: ×400.

We next evaluated the lumbar spinal cords for pathological changes in EAE mice following different treatments on day 20 p.i.. Hematoxylin and eosin staining and solochrome cyanin staining were performed for evaluating inflammatory infiltration and demyelination respectively. Histological scores of the degree of inflammation and demyelination in the lumbar spinal cord of each animal were evaluated blindly, using a semiquantitative system as previously described [17]–[20].The inflammatory scores which indicated the diffuse infiltration by mixed macrophages, T and B lymphocytes into CNS white matter in the lumbar spinal cords of the PBS- and berberine-treated EAE mice were 2.67±0.17 and 1.50±0.18 respectively. Compared with PBS-treated EAE mice, berberine treatment drastically reduced inflammatory cell infiltration in EAE mice (P<0.01, Fig. 2). Solochrome cyanin staining showed the demyelinating scores of the PBS- and berberine-treated EAE mice were 2.83±0.21 and 1.83±0.20 respectively. The demyelination in the lumbar spinal cords of EAE mice was profoundly ameliorated by berberine treatment when compared with PBS treatment (P<0.01, Fig. 2). The representative sections depicting the inflammatory infiltrates and demyelination in the lumbar spinal cords of EAE mice treated by PBS and berberine were showed in Fig 3. Inflammatory infiltrates were widespread in the PBS-treated mice at day 20 p.i. (Fig. 3A) but reduced in berberine-treated mice (Fig. 3C). And a large plaque of demyelination was seen in the PBS-treated EAE mice at day 20 p.i. (Fig. 3B), but in the berberine-treated EAE mice, demyelination was markedly attenuated (Fig. 3D).

### Berberine reduced BBB dysfunction in EAE mice

**Figure 4:**
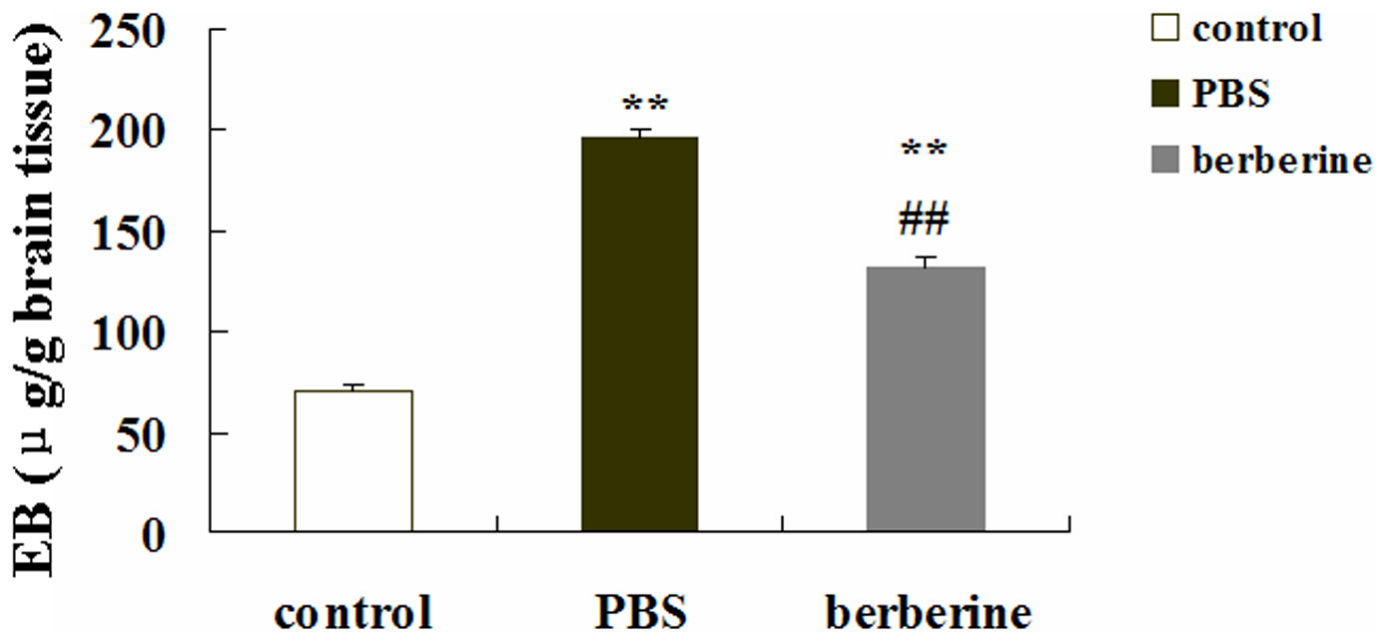
The effect of berberine on brain BBB permeability in EAE mice. The integrity of BBB was detected by quantitative measurement for EB content at day 20 p.i. in six animals per group. EB is expressed as micrograms per gram of brain tissue against a standard curve. Values represent the mean±S.E.M., ** P<0.01 vs. control mice, ^##^P<0.01 vs. PBS-treated EAE mice.

The integrity of BBB of different treated animals was detected by quantitative measurement for EB content at day 20 p.i.(n = 6). 30 minutes after the intravenous injection of EB, animals were carefully perfused to remove excess dye and the content of EB in the brain tissue was measured at 610 nm using a spectrophotometer. The concentrations of extravasated EB (expressed as micrograms per gram of brain tissue) of different groups are shown in Fig. 4. In the control mice, baseline level of EB was 70.31±3.54 µg/g brain tissue. The content of EB in the brain of PBS- and berberine-treated EAE mice were 196.70±3.76 and 130.75±5.61 µg/g brain tissue, respectively. Both PBS- and berberine-treated EAE mice showed a significant increase in the content of EB in the brain when compared with the control mice (P<0.01). But the increase in the content of EB in the brain of berberine-treated EAE mice was not as remarkable as that of the PBS-treated EAE mice (P<0.01). The results showed that berebrine could prevent further injury of BBB permeability.

### Berberine down-regulated the expression of MMP-9 but not MMP-2 in the brain of EAE mice

**Figure 5:**
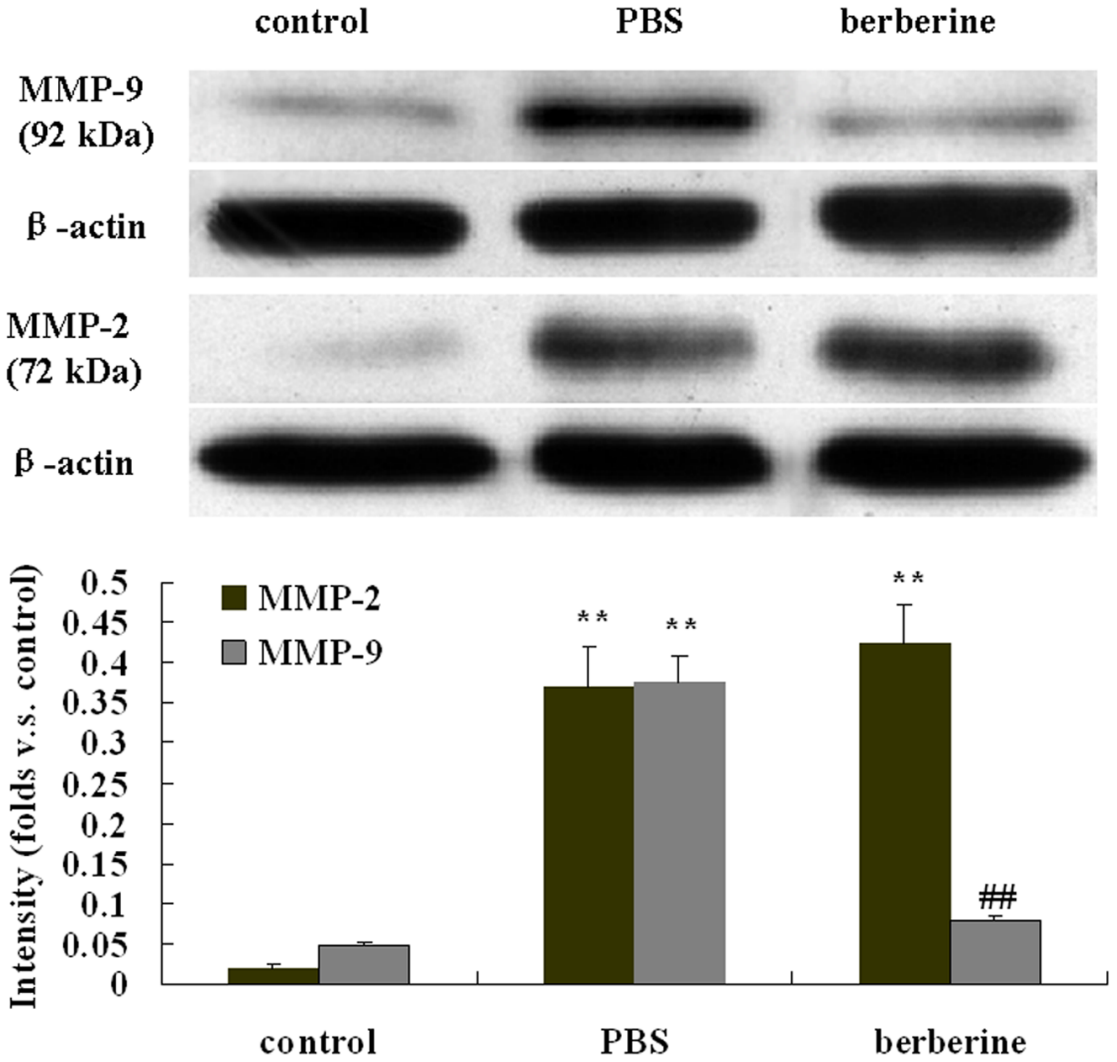
Effects of berberine on the expression of MMP-2 and MMP-9 in the brain of different treated mice. The brains from different treated mice were harvested for Western blot at day 20 p.i.. Each group had seven animals. Values represent the mean± S.E.M., **P<0.01 vs control mice, ^##^P<0.01, vs PBS-treated EAE mice.

The brains of control mice, PBS- and berebrine-treated EAE mice were obtained at day 20 p.i. (n = 7). The expression of MMP-2 and MMP-9 in the brains of different treated mice were detected by Western blot. The bands were quantified with the Quantity one image analysis software. A significant increase of MMP-2 and MMP-9 expression in the brain of PBS-treated EAE mice was recorded compared with that of the control mice (P<0.01). The increase of MMP-9 was significantly attenuated in animals treated with berberine when compared with PBS (P<0.01). However, the expression of MMP-2 in the brain of PBS- and berberine-treated EAE mice didn't show significant difference. (P>0.05, Fig. 5).

### Berberine inhibited MMP-9 activity but not MMP-2 in CSF

**Figure 6:**
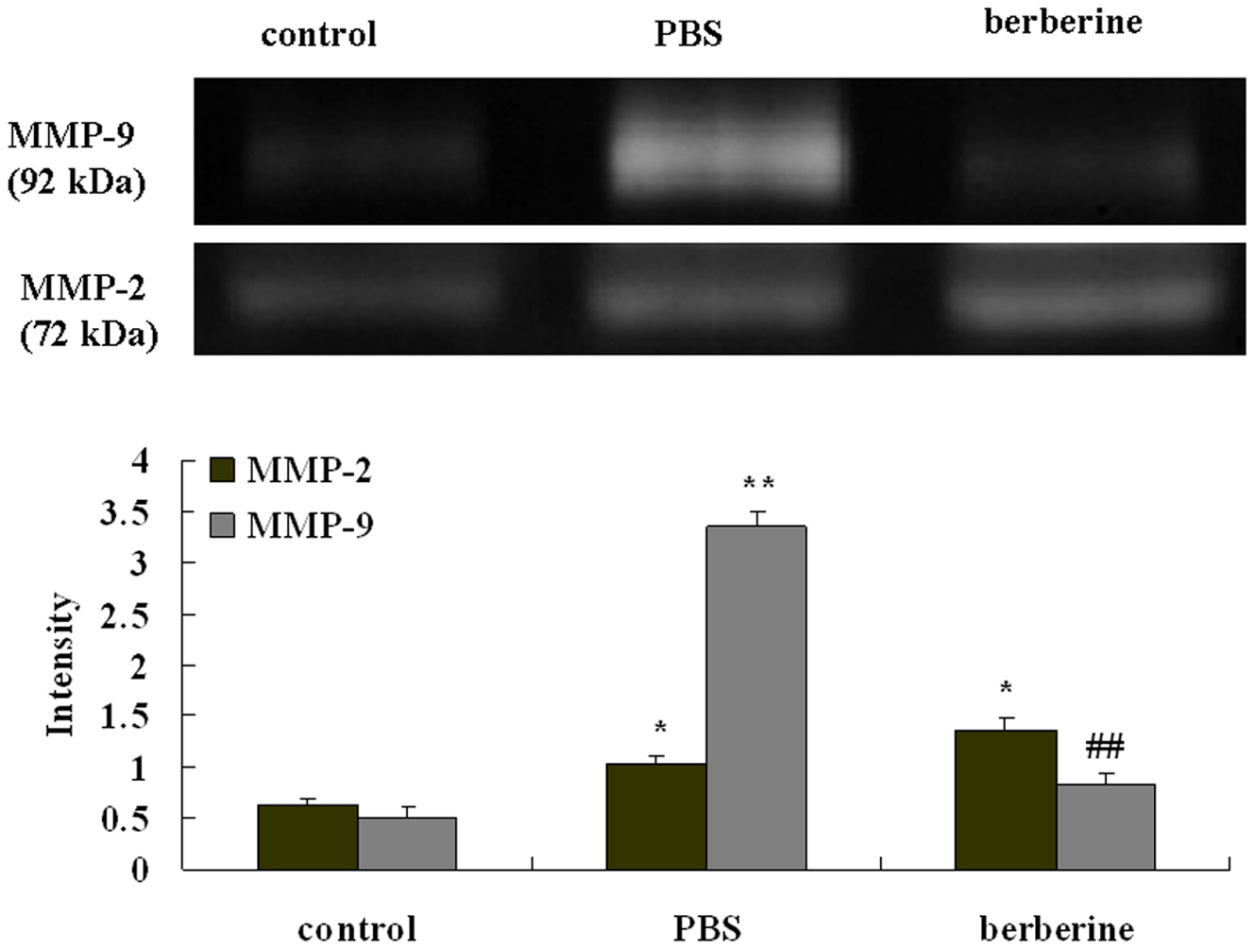
The gelatinolytic activities of MMP-2 and MMP-9 in the CSF of different treated mice. CSF was obtained by puncture of the cisternamagna to detect gelatinase activity at day 20 p.i.. Each group had seven animals. Values represent the mean± S.E.M., **P<0.01, *P<0.05 vs control mice,^ ##^P<0.01 vs PBS-treated EAE mice.

CSF of control mice, PBS- and berebrine-treated EAE mice were obtained by puncture of the cisternamagna (n = 7). To determine the activities of MMP-2 and MMP-9 in CSF, zymography was performed. The changes in MMP-2 and MMP-9 activity were well in line with the expression of protein in the brain. There was a marked increase of MMP-2 and MMP-9 activity in CSF in PBS-treated EAE mice compared to the control mice (P<0.05 for MMP-2 and P<0.01 for MMP-9, respectively). Treatment with berberine reduced MMP-9 zymographic activity in CSF compared to PBS-treated EAE mice (P<0.01). There was not significantly difference of the activity of MMP-2 between the PBS- and berberine-treated EAE mice. (P>0.05, Fig. 6).

## Discussion

Berberine has been used in the treatment of various infectious disorders in Chinese traditional medicine for more than 3000 years. However, little is known about the effects of berberine on immune inflammatory diseases such as EAE, an established model of MS. In this study, we observed that oral berberine administration initiated at the disease onset is effective to reduce the severity of EAE in C57 BL/6 mice. Within the first 3 days of berberine treatment, the neurological deficits were stabilized and clinical scores of berberine-treated EAE mice were significantly lower in comparison to the PBS-treated EAE mice. And the inflammatory infiltration and demyelination in the lumbar spinal cords of EAE mice were ameliorated by berberine treatment. These results show berberine treatment is effective to reduce the severity of EAE in C57 BL/6 mice either when evaluated clinically or by neuropathological criteria.

We also found the permeability of BBB increased in PBS-treated EAE mice, which coincided with previous reports [21]–[23]. Enhanced BBB permeability accompanying with large numbers of leucocytes infiltrating into CNS plays an important role in the development and progression of MS and EAE [24]. Transport studies using radiolabeled mannitol as a marker for the transport of compounds across the BBB revealed a 2-fold increase of transport at day 14 p.i., when the clinical signs of EAE were most abundant [25]. And various animal studies on EAE suggested that the disease severity could be correlated with alteration of BBB integrity, whereas limitation of the disease could be achieved by preventing BBB alterations in EAE [26], [27]. In our experiment, berberine treatment reduced the permeability of BBB. Reduced BBB permeability may suggest decreased leukocyte infiltration into CNS then consequently reduced CNS inflammation, which was in coincidence with the results of our histological evaluation in berberine-treated EAE mice.

In MS, large numbers of leucocytes infiltrate through BBB to the CNS in the absence of any apparent infection to produce widespread tissue insult [23], [28]. BBB permeability has been well documented to be increased by MMPs, which control cell migration across the BBB by disrupting the subendothelial basement membrane and other components of the extracellular matrix and eventually affecting tissue destruction in MS [4], [29]. Among MMPs, in particular the gelatinases MMP-2 and MMP-9 seem to be involved in mechanisms of T cell migration into the CNS and the disruption of the BBB [4], [5]. MMP-2 and MMP-9 are secreted as proenzymes (72 kDa and 92 kDa for MMP-2 and MMP-9 respectively) into the extracellular matrix, in which they are then activated by proteolytic cleavage of the amino-terminal domain (65 kDa and 84 kDa for MMP-2 and MMP-9 respectively) [30]–[32]. MMP-2 is a constitutively expressed in the latent form (72 kDa) and it is normally present in brain tissue and CSF [33]. Because of its tight association with extracellular matrix proteins, CSF 84 kDa active form of MMP-9 has been found by zymography only in a small proportion of MS [31], and active MMP-9 may have been present in the 92-kDa band because a latent metalloproteinase enzyme can be transformed into an active configuration without autocleavage of its propeptide domain, and the 84-kDa active form of MMP-9 could be coupled with increased 92- kDa gelatinolytic band intensity [31], [34], [35]. So MMP-2 and MMP-9, both in latent form, can be easily detected in the brain and CSF, and can specifically degrade type IV collagen, a key structural component of the basement membrane that surrounds blood vessels which induces the destruction of BBB [30], [31], [36].

In our experiment, we found the expression of MMP-2 and MMP-9, both in latent form, increased in the brain of PBS-treated EAE mice, and the changes in MMP-2 and MMP-9 activity in CSF were well in line with the expression of protein, which are coincided with previous reports [37]. Berberine treatment decreased the activity and expression of MMP-9 but not MMP-2 in the brain and CSF in our experiment. The results were not much coincided with previous reports. It has been demonstrated that berberine could suppress the MMP-2 expression in A 547 cells (a human non-small cell lung cancer cells with a highly metastatic capability) and inhibit MMP-9 expression in phorbol 12-myristate 13-acetate (PMA)-induced macrophages[10], [38], and berberine also could suppress migration and invasion of human SCC-4 tongue squamous cancer cells through the inhibition of MMP-2 and MMP-9 [15]. That is, berebrine could inhibit both MMP-2 and MMP-9 expression in vitro.

The possible explanation for this discrepancy may be that berberine could produce different effects on MMPs in different pathological conditions. In vivo, MMP-2 is constitutively expressed and found in healthy brain and CSF, and this constraint controls the extent of damage to the extracellular matrix of BBB [39]. However, MMP-9 is normally present at low concentrations, but rapidly increases in quantity to inflammatory stimuli such as EAE. Further more, MMP-9 is secreted into the extracellular space where it can move around freely, causes more extensive damage to the injury site of BBB [39]. Athough both MMP-2 and MMP-9 were involved in mechanisms of T cell migration into the CNS and the disruption of the BBB [30], [31], the predominant production of MMP-9 in the brain and CSF may be more effective in correlation with BBB injury in vivo while MMP-2 only exhibits a low responsiveness to inflammatory stimuli [40], [41].

It has been reported that direct injection of MMP-9 into the brain of rats induced BBB breakdown and loss of myelin staining, both of which are typical features of MS [42]. And MMP-9 activity is associated with short duration relapsing and active disease [29], [43]. MMP-9-null mice exhibit decrease of the severity of EAE [44]. However, researchers found that binding of T cells to endothelial cells could induce the expression of MMP-2 [45], particularly during the repairing processes. MMP-2 may prevail by remodeling the extracellular matrix, which may correspond with the chronic progressive stage of MS [29]. And MMP-2-null mice exhibit severer EAE with enhanced lymphocyte transmigration [46]. As a plant ingredient with anti-inflammatory effects, bererine probably achieves the effect through the decrease of the expression of MMP-9. Because an overexpression of MMP-2 could be protective in remodeling the extracellular matrix and promote tissue repair [45], [46], the benefit of berberine also may partly came from the MMP-2, because MMP-2 has not been reduced by berebrine treatment in our study.

Our results demonstrated that berberine could reduce the permeability of BBB and suppress the increased expression of MMP-9 in the brain and CSF of EAE mice. Since predominant localization of MMP-9 around blood vessels [30] suggests that MMP-9 might be pathologically involved in the disruption of the vascular basement membranes and the BBB, paving the way for infiltrating cells of the immune system [30], we can tentatively suggest that the protectetive effect of berberine on BBB is associated with a down-regulation of MMP-9 expression and then the inhibition of leucocytes infiltrating into CNS. However, recently research found that berberine can exert immunosuppressive effect through inhibiting the activation and proliferation of T cells in healthy human beings [47]. Whether berberine may have direct effects on lymphocytes or other leukocytes in EAE mice need further explore. And more studies should be taken to uncover the other mechanisms by which berebrine could improve the EAE.

Our data demonstrate that berberine is effective to attenuate the clinical severity of EAE mice. Through decreasing the expression and activity of MMP-9, berberine could protect the integrity of BBB and decrease the inflammatory infiltration. These findings suggest berberine might be a potential therapeutic agent for MS.

## Materials and Methods

### Animals and Regents

Six to eight-week-old female C57 BL/6 mice weighting 16 to 18 g were obtained from the Experimental Animal Center of Sun Yat-sen University (Guangzhou, China). Experiments were carried out according to the National Institutes of Health Guide for Care and Use of Laboratory Animals and were approved by the Bioethics Committee of Sun Yat-sen University. Berberine and complete Freund's adjuvant (CFA) were purchased from Sigma-Aldrich (St. Louis, MO). MOG35–55 peptide (MEVGWYRSPFSRVVHLYRNGK) was synthesized by CL. Bio-Scientific CO., LTD. (Xi'an, China). Amino acid sequences were confirmed by amino acid analysis and mass spectroscopy. The purity of the peptide was greater than 95%. Mycobacterium tuberculosis H37RA was purchased from Difco (Detroit, MI). Pertussis toxin (PTX) was purchased from Alexis Corp (San Diego, CA). Monoclonal anti-MMP-2 and polyclonal anti-MMP-9 were purchased from Thermo Fisher Scientific (CA, USA) and Abcam (MA, USA) respectively.

### Induction and evaluation of EAE

EAE was induced in six to eight-week-old female C57 BL/6 mice by the procedure which had been described previously [16]. Briefly, mice were immunized subcutaneously in the flanks with 200 mg of MOG35-55 peptide per animal emulsified in complete Freund's adjuvant (CFA) containing 500 µg of Mycobacterium tuberculosis H37RA. Immediately thereafter, and again 48 h later, the mice received an intraperitoneal injection of 300 ng of Pertussix toxin in 100 µl of PBS. An additional injection of MOG35–55 peptide in CFA was delivered 7 days later. The animals were examined daily for disability. Clinical scores were defined as follows: 0, no signs; 1, loss of tail tonicity; 2, flaccid tail; 3, ataxia and/or paresis of hindlimbs; 4, complete paralysis of hindlimbs; 5, moribund or death. The maximum disease score was achieved by each mouse over the course of the entire experiment. The overall disease burden of each mouse was represented as the cumulative disease severity, which was the sum of the disability scores obtained daily over the course of the 35-day experiment.

### Treatment of mice

Mice were randomly assigned to three groups: control mice, PBS-treated EAE mice, berberine-treated EAE mice. Each group has seven animals and every experiment was repeated three times. The dose of berberine was chosen on the basis of previous in-vivo data and our preliminary dose-finding experiment [48], [49], [50]. Berberine was dissolved in PBS and was intragastric administration via per os (p.o.) using a 20 ga straight oral gavage needle for mice at a dose of 30 mg/kg to EAE mice. PBS-treated EAE mice were intragastric administration PBS only. Treatment was initiated at the disease onset and medication was administered daily until mice were sacrificed at day 35 p.i.. To evaluate the treatment effects, the lumbar spinal cords of different treated EAE mice were subjected to histopathology assay. The integrity of BBB was detected by quantitative measurement for EB content. CSF was obtained by puncture of the cisternamagna to detect gelatinase activity and brains of different treated mice were prepared for Western blot to determine the expression of MMP-2 and MMP-9 at day 20 p.i..

### Histological evaluation

Histological evaluation was performed on paraformaldehyde fixed, paraffin-embedded sections of lumbar spinal cords of different treated EAE mice (n = 6). Paraffin sections were stained with hematoxylin and eosin and solochrome cyanin impregnation for evaluating inflammatory infiltration and demyelination respectively. Histopathological examination was performed in a blinded fashion. The scale evaluated for inflammation was as follows [17], [18]: 0, no inflammatory cells; 1, a few scattered inflammatory cells; 2, organisation of inflammatory infiltrates around blood vessels; 3, extensive perivascular cuffing with extension into adjacent parenchyma, or parenchymal infiltration without obvious cuffing. Demyelination in the spinal cords was scored as previously described [19], [20]: 1, traces of subpial demyelination; 2, marked subpial and perivascular demyelination; 3, confluent perivascular or subpial demyelination; 4, massive perivascular and subpial demyelination involving one half of the spinal cord with presence of cellular infiltrates into CNS parenchyma; 5, extensive perivascular and subpial demyelination involving the whole cord section with presence of cellular infiltrates into CNS parenchyma.

### Evaluation of BBB disruption

The integrity of BBB was detected by quantitative measurement for EB content at day 20 p.i. in six animals per group [51], [52]. Sterilized 2% EB (Sigma, USA) solution was administered intravenously at a dosage of 4 ml/kg per animal. 30 minutes after injection, mice were perfused with saline to remove intravascular EB dye. Brains were rapidly removed and each sample was weighed and then homogenized with 2.5 ml PBS and mixed with 2.5 ml 60% trichloroacetic acid to precipitate protein. The samples were centrifuged for 30 min at 1000 *g* and the supernatants were measured at 610 nm for absorbance of EB by using a spectrophotometer (Genesis 10uv; Thermo Electron Corporation, Madison, WI). EB is expressed as micrograms per gram of brain tissue against a standard curve.

### Western blot

To investigate the protein expression of MMP-2 and MMP-9 in the brains of the control mice, PBS- and berberine-treated EAE mice, we performed Western blot analysis. Samples of the brain from different treated mice were loaded on 10% gradient sodium dodecyl sulfate-polyacrylamide gels (20 µg protein per lane). Proteins were transferred onto nitrocellulose membrane (Bio-Rad). The membranes were blocked by 5% non-fat milk. Afterward, the membranes were incubated with monoclonal anti-MMP-2 (1∶600) and polyclonal anti-MMP-9 (1∶400) overnight respectively. After 3 times washes with TBST buffer, the membrane was incubated with anti-mouse-HRP and goat anti-rabbit-HRP for 30 minuates, respectively. The experiment was repeated in triplicate and β-actin was used as internal control. The bands were quantified with the Quantity one image analysis software.

### Gelatinase activity detection

Gelatinase activity of MMP-2 and MMP-9 in CSF was measured by sodium dodecyl sulfate-polyacrylamide gel electrophoresis zymography (SDS-PAGE) [53], [54]. After quantified with bicinchoninic acid assay (BCA) method, 10 µg of total proteins were used for each sample. A protein marker standard was used to identify molecular weights. The electrophoresis was carried out at 20 mA constant current for 2 h under non-reducing conditions in 10% polyacrylamide-SDS gels containing 1 mg/ml gelatin as the proteinase substrate [55]. The gel was washed in 50 ml of 2.5% Triton X-100 twice for 30 minutes at room temperature to remove SDS and then incubated in enzyme buffer containing CaCl_2_-Tris-NaCl for 24 h at 37°C to allow proteolysis of the gelatin substrate, fixed, and stained with Coomassie blue. The bands were quantified by densitometric analysis with Quantity one software.

### Statistical analysis

Data are expressed as means±S.E.M. and analyzed by SPSS 13.0 software. Differences between clinical scores and histological scores were analyzed with Mann-Whitney tests. Differences between gelatinase activity, protein levels and EB content were analyzed with One-Way ANOVA followed by Bofferroni's post hoc tests. *P* values less than 0.05 were considered statistically significant.
